# Electric stimulation of the ears ameliorated learning and memory impairment in rats with cerebral ischemia-reperfusion injury

**DOI:** 10.1038/srep20381

**Published:** 2016-02-05

**Authors:** Ching-Tung Kuo, Yi-Wen Lin, Nou-Ying Tang, Chin-Yi Cheng, Ching-Liang Hsieh

**Affiliations:** 1 Graduate Institute of Acupuncture Science, College of Chinese Medicine, China Medical University, Taichung 40402, Taiwan; 2 School of Chinese Medicine, College of Chinese Medicine, China Medical University, Taichung 40447, Taiwan; 3 Graduate Institute of Integrated Medicine, College of Chinese Medicine, China Medical University, Taichung 40402, Taiwan; 4 Department of Chinese Medicine, China Medical University Hospital, Taichung 40447, Taiwan; 5 Research Center for Chinese Medicine and Acupuncture, China Medical University, Taichung 40402, Taiwan

## Abstract

Ear acupuncture enhances the secretion of acetylcholine, which has anti-inflammatory effects. Here we want to investigate the effect of electric stimulation (ES) of the ears on learning and memory impairment in rats with cerebral ischemia-reperfusion injury. At 24 h after reperfusion, 2-Hz ES was applied to the ears for 20 min/day (10 min for each ear) for 7 days continuously. The step-through time of the passive avoidance test was greater in the ES group than in the control group (300.0 ± 0.0 s vs 45.0 ± 26.7 s, *p* < 0.05). Our results showed that neither neurological deficit score nor motor functions were improved after 2-Hz ES (4.0 ± 0 vs 4.5 ± 0.8, *p* > 0.05). The numbers of nicotinic acetylcholine receptor α4 positively stained cells in the CA2 and dentate gyrus of the hippocampus were 19.0 ± 11.5 and 269.2 ± 79.3, respectively, in the ES group, which were greater than those in the control group (7.0 ± 5.9 and 165.5 ± 30.8, respectively) (both *p* < 0.05). These results suggested that 2-Hz ES of the ears ameliorated learning and memory impairment in rats with ischemia-reperfusion injury. ES of the ears has neuroprotective effects, which are related to acetylcholine release.

Stroke is the second most common cause of death worldwide^1^, and approximately 6.5 million stroke patients die each year; however, 25.7 million patients survive^2^. Ischemic stroke, the major cause of stroke, accounts for approximately 80% of deaths^3^. Dementia prevalence in stroke survivors is approximately 30%, and the incidence rate of dementia is 7% 1 year after stroke and gradually increases to 48% 25 years after stroke^4^. Dementia is mainly characterized by cognitive function decline in one or more domains, including learning and memory, language, executive function, complex attention, perceptual-motor, and social cognition^5^. Nicotinic acetylcholine receptors (nAChRs) play a critical role in maintaining cognitive function; however, the expression of nAChRs declines with age and in dementia^6^,^7^,^8^. Therefore, the functioning of nAChRs exerts neuroprotective effects against neurodegenerative diseases and prevents cognitive function decline. Ki67 has been reported as an effective mitotic marker because of its expression in all cell cycle phases except the resting phase^9^.

Several studies have reported that ear stimulation increases parasympathetic activity^10^,^11^, and it has been hypothesized that auricular acupuncture increases parasympathetic activity, and the subsequent activation of the cholinergic anti-inflammatory pathway controls inflammation, thereby reducing epilepsy^12^. Our previous study found that 2-Hz electroacupuncture at Baihui (GV20) can restore long-term potentiation, which is closely associated with memory in rats with cerebral ischemia-reperfusion injury^13^. Therefore, we hypothesize that electric stimulation of the ears can increase neuronal nAChRs to ameliorate learning and memory impairment, and established a learning and memory impairment rat model through transient middle cerebral artery occlusion (MCAo) followed by reperfusion. Electric stimulation (ES) at 2-Hz was applied to the ears, and the behavior of the rats was observed using the passive avoidance test.

## Materials and Methods

### Experimental animals

The present study used male Sprague–Dawley (SD) rats, weighing 280–310 g, purchased from BioLASCO Taiwan Co., Ltd., and these rats were raised in the Animal Center of China Medical University (CMU) in accordance with the Guide for the Care and Use of Laboratory Animals. A 12:12 h light–dark cycle was applied, room temperature was controlled at 25 °C, and the rats were provided adequate food and water. The study protocol was approved by the Institutional Animal Care and Use Committee of CMU.

### Induction of ischemia-reperfusion injury in the learning and memory impairment rat model

Cerebral ischemia was induced by intraluminal suture occlusion of the middle cerebral artery (MCA), as described previously^14^. The rats were anesthetized with isoflurane (Aerrane, Canada) by using a vaporizing system (MATRX VIP 3000; Midmark, USA) and then placed in a stereotaxic apparatus in the prone position. The right parietal bone was thinned using a grinding machine to monitor the blood flow. Laser Doppler flowmetry probe was placed 5 mm lateral, 1 mm posterior to the bregma to measure the blood flow of the right MCA. We set Laser Doppler flowmetry values as a monitor during the ischemia process. Values over 10 min before ischemia were averaged and set as baseline (100%). We include the rats for further experiment since the values less than 50%.

The rats were then placed in the supine position, and the neck was incised from the midline to expose the right common carotid artery. The right internal and common carotid arteries were clipped, and the external carotid artery was permanently ligated, and an incision was made. A 3-0 nylon filament suture, the tip of which was smoothed by heat and coated with poly-L-lysine (UNIK, Taiwan), was advanced for a distance of approximately 23–25 mm from the incision of the external carotid artery through the common carotid artery into the internal carotid artery to block the origin of the MCA. The blood flow of the right MCA was monitored using the Laser Doppler Blood-Flow Monitor (DRT4, Moor Instrument Ltd., England), and when the marked scale on the screen declined from 300 to 50, blockage of cerebral blood flow was confirmed. In the present study, the cerebral blood flow of the right MCA was blocked for 10 min and then restored.

### Neurological status evaluation

The neurological status was evaluated by a well-trained person who was blinded to the groups according to the Modified Neurological Severity Score, as described by Chen *et al.*^15^, at 24 h (first day) and on the seventh day after reperfusion. The total neurological deficit score is 18 and comprises motor test (raising the rat by the tail, 0–3; placing the rat on the floor, 0–3), sensory test (0–2), beam balance test (0–6), and reflex test (0–4) scores.

### Grouping

In the present study, only rats with a Modified Neurological Severity Score higher than 5 were included. A total of 18 SD rats were randomly divided into three groups as follows: (1) sham group (SG): the common carotid artery of rats was exposed without blocking the cerebral blood flow of the MCA, and rats did not undergo ES but were anesthetized for 20 min/day for 7 days continuously 24 h after surgery; (2) control group (CG): the methods were identical to those applied for the SG, but the cerebral blood flow of the right MCA was blocked for 10 min and subsequently restored; (3) ES group: the methods were identical to those used for the CG; however, ES (frequency: 2 Hz, intensity: 2 mA, duration: 100 μs) was applied to the ears (cathode in the apex and anode in the lobe) for 20 min/day (10 min for each ear) for 7 days continuously 24 h after reperfusion by using an ES apparatus (Trio 300, Ito, Japan).

### Passive avoidance test

After neurological status evaluation, the passive avoidance test was performed using the Gemini Avoidance System (San Diego Instruments, San Diego, CA, USA), which consists of two identical chambers (25 × 20 × 17 cm) connected by a gate (9 × 7 cm). The floor of each chamber is composed of 14 stainless steel rods (6 mm in diameter), with an interval of 1.8 cm, that are connected to a shock scrambler. The passive avoidance test was performed as follows.

#### Habituation: 1 day before right MCAo

The rats were placed in the right chamber (bright room) of the system; 5 s later, the right chamber was illuminated, and the gate opened simultaneously. Once the rats entered the left chamber (dark room), the gate closed instantaneously, and the entrance latency was recorded. After 30 s, the rats were shifted from the dark room to the rat cage. If the rats stayed in the bright room for more than 100 s, they were excluded from the experiment. After 30 min, the habituation session was repeated, and the maximum latency was selected.

#### Training: 1 h before MCAo

The procedure was similar to that of the habituation session. After rats entered the dark room, the gate closed immediately, intermittent electric shocks (50 Hz, 3 s, 0.5 mA) were delivered, and the number of training sessions was recorded. After 30 s, the rats were transferred back to the rat cage. The latency was recorded as 120 s if the rats did not enter the dark room. After 2 min, the aforementioned process was repeated once, and the longest latency (in seconds) that occurred first was selected. The process was repeated again after 2 min.

#### Retention trial: 24 h and on the seventh day after reperfusion

The procedure was similar to that of the training session. If the time for which the rats stayed in the bright room [step-through latency (STL)] was greater than 300 s, the STL was recorded as 300 s, whereas if the rats stayed in the bright room for less than 300 s, the STL recorded was the actual stay time. The retention trial was performed twice 24 h after reperfusion and three times on the seventh day after reperfusion. The longest STL was selected.

### Rotarod test

After the retention trial, the rats were placed on a Rotamex (Columbus Instrument, Ohio, USA) with an initial speed of 4 rpm and which increased by 1 rpm every 8 s until the maximum speed of 40 rpm was attained. The time spent by the rat on the Rotamex before falling was recorded, the test was performed six times, and the average of the three longest times recorded was calculated.

### Immunohistochemical staining

After the rotarod test, the rats were anesthetized using chloral hydrate (400 mg/mL), and then perfused with 200 mL of 0.9% saline, and their brains were removed. The brains were fixed in 4% paraformaldehyde (pH 7.4) for 3 days and were transferred to 30% sucrose (w/v) for 3 days for dehydration. The brains were embedded in FSC 22 Frozen Section Media (Leica Surgipath, USA) and were frozen solid. The frozen brains were cut into 16-μm sections in CM3050S (Leica, USA), and these sections were mounted on mounting media (Assistant-Histokitt, Germany).

The brain sections mounted on a slide were rinsed with 0.01% Tween 20/phosphate buffered saline (PBS) twice and soaked in 3% H_2_O_2_/methanol for 15 min to inhibit endogenous peroxidase activity as an inset. Sections were then incubated with 10% normal animal serum (A kit) (Zymed, CA, USA) for 20 min at room temperature.

The sections were incubated with a primary antibody, the anti-GFAP antibody (1:200), for 30 min at room temperature. Subsequently, sections were incubated with other primary antibodies, the nicotinic acetylcholine receptor α4 (nAChRα4) (1:500), Ki67 (1:100), and NeuN antibodies (1:1000), for 1 day at −4 °C in a moisture chamber. After incubation with secondary antibodies (B kit) and the avidin–biotin peroxidase complex (C kit) (Zymed), the sections were colored using a 3,3-diaminobenzidine kit (Scytek Laboratories, Logan, UT, USA) and counterstained with hematoxylin. The stained sections were embedded in mounting media (Assistant-Histokitt, Germany), and the number of immunoreactive cells was calculated under the Axioskop 40 microscope (Zeiss, Germany). In addition, PBS was used as a substitute for primary antibodies in the negative control, which was subjected to the same immunohistochemical assay procedures. Six rats in total were employed, and 20 slides were captured for each group.

### Statistical analyses

Data are presented as the mean ± standard deviation. The data of the SG, CG, and ES group were compared using one-way analysis of variance, followed by Tukey’s test, using the Origin7.5 analysis statistical software. A *p* value less than 0.05 was considered statistically significant. The sample size required for an alpha of 0.05 and a power of 80% is 6 cases for one group.

## Result

### Effects of ES of the ears on neurological deficit score in rats with cerebral ischemia-reperfusion injury

The neurological deficit scores in the CG and the ES group were higher than those in the SG at 24 h and on the seventh day after reperfusion (all *p* < 0.05; Table 1). No significant differences were observed in the neurological deficit scores in the CG and the ES group at 24 h and on the seventh day after reperfusion (both *p* > 0.05; Table 1).

### Effects of ES on STL in the passive avoidance test in rats with cerebral ischemia-reperfusion injury

No significant differences were observed in the STL in the passive avoidance test between two groups among the SG, CG, and ES group at 24 h and 1 h before MCAo and at 24 h after reperfusion (all *p* > 0.05; Table 2). The STL in the CG was lower than that in the SG and ES group (both *p* < 0.05; Table 2). No significant differences were observed in the STL in the SG and ES group on the seventh day after reperfusion (*p* > 0.05; Table 2).

### Effects of ES on rotarod test times in cerebral ischemia-reperfusion injured rats

The times recorded for the rotarod test on the seventh day after reperfusions were 57.9 ± 26.1 s in the SG, 71.1 ± 11.5 s in the CG, and 67.6 ± 50.3 s in the ES group. No significant differences were observed between two groups among the SG, CG, and ES group (all *p* > 0.05).

### Effects of ES on numbers of nAChRα4, Ki67, GFAP and NeuN positively stained cells in rats with cerebral ischemia-reperfusion injury

**Figure 1:**
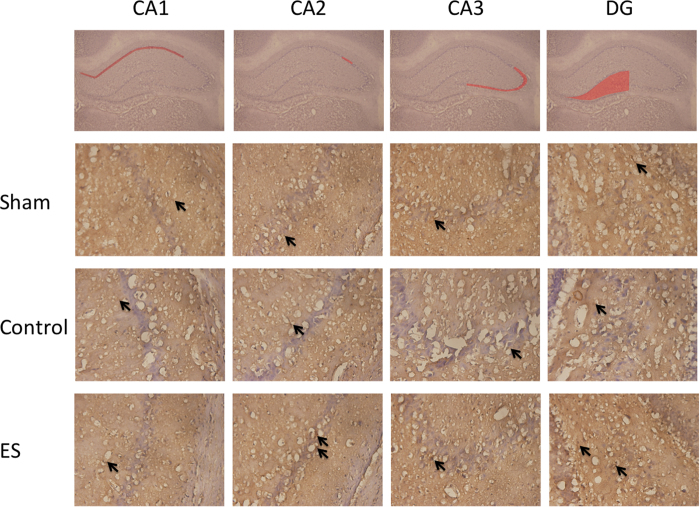
Effect of 2-Hz electric stimulation on number of nicotinic acetylcholine receptor α4 (nAChR α4) positively stained cells (400×). Sham: sham group; Control: control group; ES: electric stimulation group. In CA2 and the DG, the number of nAChR α4 positively stained cells (arrow) in the ES was greater than those in the Sham and Control. DG: dentate gyrus.

In the CA2 and DG regions of the hippocampus, the numbers of nAChR α4 positively stained cells in the ES group were greater than those in the SG and CG (all *p* < 0.05; Table 3; Fig. 1), whereas no significant differences were observed between the SG and CG (both *p* > 0.05; Table 3; Fig. 1).

In the CA1 and CA3 regions of the hippocampus, no significant differences were observed in the numbers of nAChR α4 positively stained cells between two groups among the SG, CG, and ES group (all *p* > 0.05; Table 3; Fig. 1).

**Figure 2:**
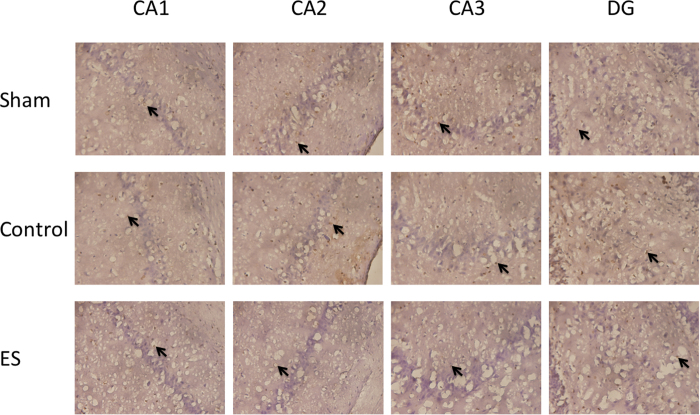
Effect of 2-Hz electric stimulation on number of Ki67 positively stained cells (400×). Sham: sham group; Control: control group; ES: electric stimulation group. In CA1, CA2, CA3, and the DG, the numbers of Ki67 positively stained cells (arrow) were similar in the Sham, Control, and ES. DG: dentate gyrus.

In the CA1, CA2, CA3, and DG regions of the hippocampus, no significant differences were observed in the numbers of Ki67 positively stained cells between two groups among the SG, CG, and ES group (all *p* > 0.05; Table 3; Fig. 2).

**Figure 3:**
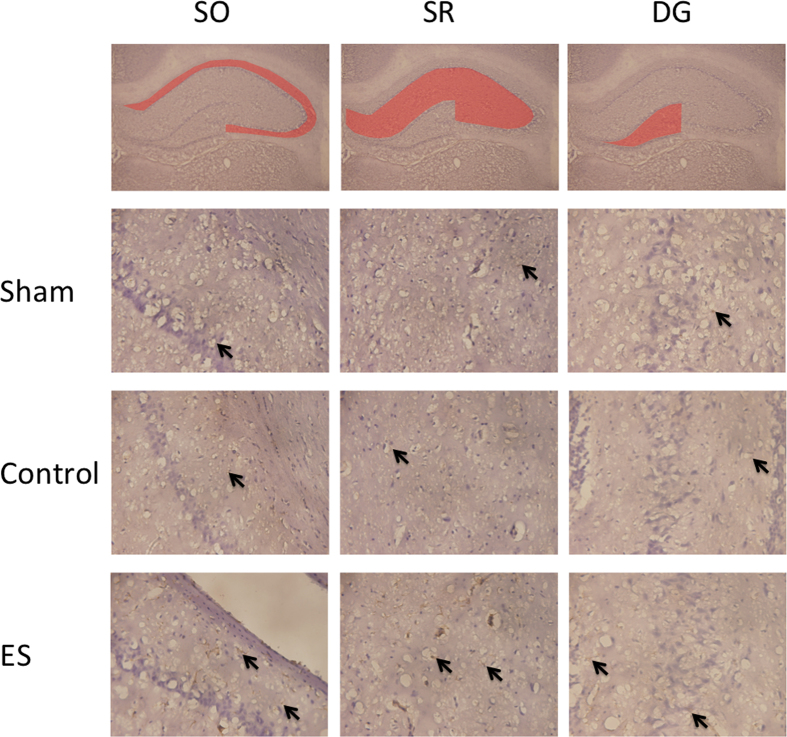
Effect of 2-Hz electric stimulation on number of GFAP positively stained cells (400×). Sham: sham group; Control: control group; ES: electric stimulation group. In the SO, SR, and DG, the number of GFAP positively stained cells (arrow) in the ES was greater than those in the Sham and Control. SO: strata oriens; SR: strata radiatum; DG: dentate gyrus.

In the SO, SR, and DG regions of the hippocampus, the numbers of GFAP positively stained cells in the ES group were greater than those in the SG and CG (all *p* < 0.05; Table 3; Fig. 3), whereas no significant differences were observed between the SG and CG (both *p* > 0.05; Table 3; Fig. 3).

**Figure 4:**
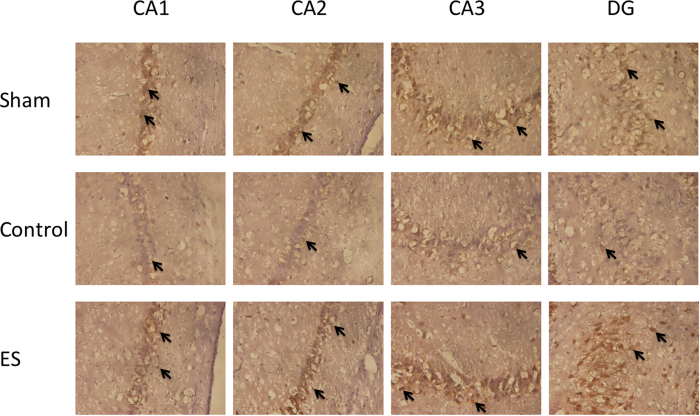
Effect of 2-Hz electric stimulation on number of NeuN positively stained cells (400×). Sham: sham group; Control: control group; ES: electric stimulation group. In CA1, CA2, CA3, and the DG, the numbers of NeuN positively stained cells (arrow) in the Sham and ES were greater than that in the Control. DG: dentate gyrus.

In the CA1, CA2, CA3, and DG regions of the hippocampus, the numbers of NeuN positively stained cells in the CG were lower than those in the SG and ES group (all *p* < 0.05; Table 3; Fig. 4), whereas no significant differences were observed in the numbers of NeuN positively stained cells between the SG and ES group (all *p* > 0.05; Table 3; Fig. 4).

## Discussion

### ES of the ears can ameliorate learning and memory impairment in rats with cerebral ischemia-reperfusion injury

The present study established an ischemia-reperfusion injury, learning and memory impairment rat model through right MCAo for 10 min, followed by reperfusion. The results indicated that no significant differences were observed in the STL in the passive avoidance test between two groups among the SG, CG, and ES group at 24 h and 1 h before MCAo and at 24 h after reperfusion. The STL in the CG was lower than that in the SG and ES group. The results suggested that learning and memory impairment was induced in the rats on the seventh day after reperfusion, and the behavior associated with learning and memory impairment could be ameliorated by ES of the ears. Vascular dementia (postinfarction dementia) is the second leading cause of dementia in older people after Alzheimer disease^16^,^17^. The exact mechanism of ischemic dementia is unclear; however, evidence indicates that a lack of choline after ischemia causes inflammation, leading to delayed brain injury^18^,^19^,^20^. The anti-inflammatory effect of choline is at least partially neuroprotective^21^. Inflammation plays a major role in the acute pathology progress of ischemic brain injury^22^ and leads to subsequent brain damage. In local ischemia, brain tissue undergoes acute softening at the early stage, which results in a pattern of alternating cell swelling and shrinkage. Inflammation is observed in the next 3–4 days, with release on the 10th day after ischemia^23^. Inflammatory mediators are released after stroke, and the permeability of the blood–brain barrier (BBB) rapidly increases, leading to a local immune response. Within a few minutes of stroke onset, white blood cells and glial cells (stellate cells and small plastic glue cells) are rapidly activated on the penumbral area. The mitochondria of these neuronal cells begin to swell extensively and release toxic substances that can damage the BBB. Astrocytes and synapses in the same lesion selectively swell, and no changes are observed in oligodendrocyte cells and capillaries at the initial stage of ischemic injury^23^,^24^,^25^,^26^. In addition, the BBB damage results in increased inflammation, causing secondary ischemic injury^27^.

### ES of the ears can increase nAChR α4 release in the hippocampus in rats with cerebral ischemia-reperfusion injury

Our results indicated that in the CA2 and DG regions of the hippocampus, the numbers of nAChR α4 positively stained cells in the ES group were greater than those in the SG and CG. These results showed that ES can increase nAChR α4 release in the hippocampus. Through the stimulation and activation of nAChRs, nicotine can improve cognitive function and has a neuroprotective role against the development of dementia^6^. In addition, acetylcholine esterase inhibitors used in cholinergic replacement therapy alleviate the symptoms of Alzheimer disease^8^.

The auricular branch of the vagus nerve is the only peripheral branch of the vagus nerve^28^. Several studies have found that auricular acupuncture regulates the endocrine system and the autonomic nervous system^29^, and can increase the activity of the vagus nerve^30^. Stimulating the vagus nerve produces an incoming signal in the brain that induces the release of acetylcholine, which reduces inflammation and apoptosis by interacting with immune cells^31^. The hippocampus plays a crucial role in memory. DG cells receive excitatory neuron input from the olfactory cortex and send excitatory neuron output to the CA3 region through mossy fibers^32^ and subsequently to CA2. Finally, the output is sent by CA1. Recently, a study showed that CA2 can enhance the input from CA3^33^. Another study reported that CA2 cell damage can lead to social memory loss^34^. Altogether, ES of the ears can increase nAChR α4 release and alleviate learning and memory impairment in rats with ischemia-reperfusion injury.

### ES of the ears could not change Ki67 protein levels but could increase astrocytes and neuronal cells in rats with cerebral ischemia-reperfusion injury

Our results indicated that in the CA1, CA2, CA3, and DG regions of the hippocampus, no significant differences were observed in the numbers of Ki67 positively stained cells between two groups among the SG, CG, and ES group. The expression of Ki67 protein is a marker of proliferating cells in the cell cycle. This antigen can be detected in the nucleus, and most of the protein is relocated to the surface of the chromosomes during the mitotic phase of the cell cycle^35^. Thus, further study of the relationship between ES of the ears and cell proliferation is required in the future.

Our results indicated that the numbers of GFAP positively stained cells in the ES group were greater than those in the SG and CG in the SO, SR, and DG regions of the hippocampus. GFAP is an intermediate filament protein, and GFAP positively stained cells may represent astrocytes in the central nervous system. Reactive gliosis and glial scar formation occur in the human brain after ischemic stroke. This gliosis may represent an increase in the lesion size and tissue damage and may function as protective barrier against infectious agents and inflammatory cell invasion^36^. Thus, astrocytes have as a dual role in brain tissue repair in the central nervous system. Astrocytes play a role in transport; for example, they can serve as glutamate transporters, reducing glutamate accumulation, astrocyte–neuron signaling, and neurotransmission. Astrocytes also play a critical role in repairing the damaged BBB after stroke and brain trauma^37^.

Our results indicated that the numbers of NeuN positively stained cells in the CG were lower than those in the SG and ES group in the CA1, CA2, CA3, and DG regions of the hippocampus. NeuN positively stained cells are neuron markers^38^; ES of the ears increased the number of NeuN positively stained cells, suggesting that it increased neuronal survival. Altogether, the findings suggest that 2-Hz ES of the ears produces a neuroprotective effect against ischemia-reperfusion injury.

In the present study, conflicting results were obtained for the CG and ES group. No significant differences were observed in the neurological deficit scores between the CG and ES group at 24 h and on the seventh day after reperfusion. In addition, no significant differences were observed in the time recorded for the rotarod test on the seventh day after reperfusion between these two groups. We suggest that because ischemia was induced for only 10 min in the present study, the ischemic infraction volume was small, and collateral circulation in the rat brain was increased. Motor function also recovers rapidly and may have recovered by the seventh day after reperfusion when both neurological status evaluation and the rotarod test were performed.

## Conclusion

ES of the ears at 2 Hz ameliorates learning and memory impairment in rats with ischemia-reperfusion injury, thus exhibiting neuroprotective effects, and these effects are related to acetylcholine release.

## Additional Information

**How to cite this article**: Kuo, C.-T. *et al.* Electric stimulation of the ears ameliorated learning and memory impairment in rats with cerebral ischemia-reperfusion injury. *Sci. Rep.* **6**, 20381; doi: 10.1038/srep20381 (2016).

## Figures and Tables

**Table 1:** Effect of electric stimulation of the ears on neurological deficit scores in rats with ischemia-reperfusion injury.

	P1	P7
Sham	0 ± 0	0 ± 0
Control	5.3 ± 0.5*	4.0 ± 0*
ES	5.2 ± 0.4*	4.5 ± 0.8*

Mean ± standard deviation. P1: neurological deficit scores at 24 h after reperfusion; P7: neurological scores at 7^th^ day after reperfusion; Sham: sham group without ischemia; Control: normal group with ischemia for 10 min and then reperfusion; ES: ES group, electric stimulation at ear start 24 h after reperfusion; *p < 0.05 compared to Sham.

**Table 2:** Effect of electric stimulation of the ears on step-through latency in the passive avoidance test in rats with ischemia-reperfusion injury (in seconds).

	H	TS	RT1	RT7
Sham	30.1 ± 14.4	14.6 ± 7.0	69.8 ± 113.7	285.4 ± 35.8
Control	13.7.1 ± 6.8	25.2 ± 33.0	69.6 ± 81.6	45.0 ± 26.7*
ES	25.9 ± 15.2	31.1 ± 43.9	177.8 ± 98.0	300.0 ± 0.0^#^

Mean ± standard deviation. H: avoidance test at 24 h prior to ischemia; TS: avoidance test at 1h prior to ischemia; RT1: avoidance test 24 h after reperfusion; TR7: at 7^th^ day after reperfusion; Sham: sham group without ischemia; Control: normal group with ischemia for 10 min and then reperfusion; ES: ES group, electric stimulation at ear start 24 h after reperfusion; *p < 0.05 compared to Sham; ^#^p < 0.05 compared to Control.

**Table 3:** Effect of electric stimulation of the ears on number of nAChR α4, Ki67, GFAP, NeuN positively stained cells in rats with ischemia-reperfusion injury.

	Sham	Control	ES
nAChR α4			
CA1	50.6 ± 29.7	57.2 ± 29.6	127.0 ± 101.0
CA2	4.7 ± 3.9	7.0 ± 5.9	19.0 ± 11.5*^#^
CA3	39.7 ± 24.3	64.5 ± 28.6	93.2 ± 50.8
DG	146.7 ± 65.3	165.5 ± 30.8	269.2 ± 79.3*^#^
Ki67			
CA1	29.5 ± 19.4	37.3 ± 10.9	40.3 ± 23.6
CA2	6.2 ± 4.8	7.2 ± 4.1	7.7 ± 3.2
CA3	32.2 ± 18.8	40.8 ± 17.2	44.0 ± 21.8
DG	286.5 ± 97.5	274.0 ± 42.3	215.7 ± 67.3
NeuN			
CA1	271.3 ± 157.1	36.3 ± 36.2	259.8 ± 151.8*^#^
CA2	38.2 ± 17.8	5.8 ± 10.6	41.2 ± 19.6*^#^
CA3	213.0 ± 55.5	85.0 ± 86.2	245.3 ± 66.4*^#^
DG	253.7 ± 107.8	83.8 ± 44.3	270.5 ± 62.7*^#^
GFAP			
SO	48.5 ± 42.3	34.0 ± 52.2	134.2 ± 63.7*^#^
SR	94.8 ± 81.6	67.3 ± 78.9	291.8 ± 98.4*^#^
DG	35.3 ± 37.0	35.2 ± 62.6	140.7 ± 84.8*^#^

Mean ± standard deviation. nAChRα4: nicotine acetylcholine receptor α4 positively stained cells; Ki67: Ki67 positively stained cells; NeuN: NeuN positively stained cells ; GFAP: GFAP positively stained cells; CA1: CA1 region of hippocampus; CA2: CA2 region of hippocampus; CA3: CA3 region of hippocampus; DG: dentate gyrus of hippocampus; SO: strata oriens region of hippocampus; SR: strata radiatum region of hippocampus; Sham: sham group without ischemia; Control: normal group with ischemia for 10 min and then reperfusion; ES: ES group, electric stimulation at ear start 24 h after reperfusion; *p < 0.05 compared to Sham; ^#^p < 0.05 compared to Control.
